# Potential rise in iron deficiency due to future anthropogenic carbon dioxide emissions

**DOI:** 10.1002/2016GH000018

**Published:** 2017-08-02

**Authors:** M. R. Smith, C. D. Golden, S. S. Myers

**Affiliations:** ^1^ Exposure, Epidemiology and Risk Program, Department of Environmental Health Harvard T.H. Chan School of Public Health Boston Massachusetts USA; ^2^ Harvard University Center for the Environment Cambridge Massachusetts USA

**Keywords:** iron deficiency, climate change, global health, nutrition

## Abstract

Iron deficiency reduces capacity for physical activity, lowers IQ, and increases maternal and child mortality, impacting roughly a billion people worldwide. Recent studies have shown that certain highly consumed crops—C_3_ grains (e.g., wheat, rice, and barley), legumes, and maize—have lower iron concentrations of 4–10% when grown under increased atmospheric CO_2_ concentrations (550 ppm). We examined diets in 152 countries globally (95.5% of the population) to estimate the percentage of lost dietary iron resulting from anthropogenic CO_2_ emissions between now and 2050, specifically among vulnerable age‐sex groups: children (1–5 years) and women of childbearing age (15–49 years), holding diets constant. We also cross‐referenced these with the current prevalence of anemia to identify most at‐risk countries. We found that 1.4 billion children aged 1–5 and women of childbearing age (59% of global total for these groups) live in high‐risk countries, where the prevalence of anemia exceeds 20% and modeled loss in dietary iron would be in the most severe tertile (>3.8%). The countries with the highest anemia prevalence also derive their iron from the fewest number of foods, even after excluding countries consuming large amounts of unaccounted wild‐harvest foods. The potential risk of increased iron deficiency adds greater incentive for mitigating anthropogenic CO_2_ emissions and highlights the need to address anticipated health impacts via improved health delivery systems, dietary behavioral changes, or agricultural innovation. Because these are effects on content rather than yield, it is unlikely that consumers will perceive this health threat and adapt to it without education.

## Key Points

- A total of 1.4 billion women and children are in countries with >20% anemia prevalence and where their iron supply could fall by >3.8% under 550 ppm CO_2_
- High‐risk regions are economically poorer and derive iron mainly from plants: S. Asia, Middle East, E. Africa, SE Asia, and N. Africa
- Countries with the highest prevalence of anemia derive their iron from the fewest foods, leaving them with less options to switch to

## 1 Introduction

Insufficient iron is the most common micronutrient deficiency in the world [*Umbreit*, 2005]. Though the full global extent of iron deficiency is unknown, nearly two billion people suffer from anemia [*World Health Organization* (*WHO*), 2011], roughly half of which is assumed to be attributable to a lack of adequate iron [*Stoltzfus et al*., 2004], the most common cause globally [*Kassebaum et al*., 2014]. Iron deficiency is also implicated in nearly 200,000 deaths and 45 million disability‐adjusted life‐years (DALYs) lost annually (4.5% of all risk‐attributable DALYs) in the most recent 2013 Global Burden of Disease Study, predominantly due to iron deficiency anemia [*Forouzanfar et al*., 2015]. Furthermore, iron deficiency in the absence of anemia has been linked to many detrimental outcomes: lowered cognitive ability, reduced work capability, and greater rate of maternal and child mortality [*Stoltzfus*, 2003].

For most people around the world, dietary iron is derived chiefly from the consumption of plants. However, there are many environmental and agronomic factors that can influence a plant's iron content, including soil management and the type of cultivar planted [*Rengel et al*., 1999; *Fan et al*., 2008; *Zhao et al*., 2009; *Pinson et al*., 2015]. Recently, *Myers et al.* [2014] demonstrated that the edible portions of food crops grown in open field conditions under elevated atmospheric CO_2_ of 550 parts per million (hereafter eCO_2_) have significantly decreased iron contents by 4–10%. These CO_2_ levels are projected to occur by roughly 2050, even if interventions are made to curb emissions [*Fisher et al*., 2007]. Specifically, C_3_ grasses (rice and wheat), legumes, and maize showed significant iron losses, while no effect was found in sorghum.

Likewise, protein and zinc also declined under eCO_2_, and companion studies to this one have examined the dietary supply and health implications for those losses [*Myers et al*., 2015; *Medek et al*., 2017]. Here we focus on the impact of anthropogenic (human‐derived) CO_2_ emissions on global dietary iron intake. The goal of this study is to look at the total effect on dietary supplies across countries and among groups that are particularly vulnerable to iron losses [*Umbreit*, 2005]: children (1–5 years) and women of childbearing age (15–49 years). Furthermore, we isolated those countries whose populations are currently most iron deficient and who are likely to see the greatest losses of iron in the diet from eCO_2_‐related effects, thereby identifying the most high‐risk regions for future intervention and study.

## 2 Materials and Methods

The objectives of our analysis were to (1) determine the dietary supply of iron in each country and also from which foods it was sourced, (2) quantify the decrease in iron content of various crops grown under eCO_2_ conditions, matched with dietary sources to predict the decrease in total iron under future eCO_2_ scenarios per capita per country, and (3) identify the countries that are most vulnerable to dietary iron losses and, of those, which have a current high prevalence of anemia. Our goal is to identify high‐risk regions where a high prevalence of contemporary iron deficiency overlaps with large vulnerability to the eCO_2_ effect. Because the change in dietary iron is “hidden” as a decline in iron content within existing foods, we believed that food substitution or replacement scenarios would be unlikely, and they were not modeled.

Food and iron supplies, defined as those available to be eaten, for the relevant age and sex groups were estimated using the Global Expanded Nutrient Supply (GENuS) model. A brief description of GENuS is provided here while a more complete description has been published [*Smith et al*., 2016]. All GENuS food and nutrient data sets are freely available online at https://dataverse.harvard.edu/dataverse/GENuS.

First, edible food supplies were estimated for 152 countries (95.5% of global population), which constitute a total of 2.4 billion people within the age‐sex groups targeted in our study: 0.6 billion children between ages 1 and 5, and 1.8 billion women of childbearing age. We estimated the supply of 225 individual foods that attempt to capture the breadth of human diets using data from Food and Agriculture Organization (FAO) food balance sheets [*Food and Agriculture Organization* (*FAO*), 2015], FAO food production and trade data, and cereal milling estimates supplied by the *Wessells et al.* [2012]. Edible food supplies were then disaggregated further by age‐sex group to study only those populations that are most vulnerable to the health burden associated with decreased iron intake: children (ages 1–5) and women of childbearing age (ages 15–49). To do this, we used the Global Dietary Database (GDD) [*Global Nutrition and Policy Consortium*, 2015], which models the differential intake of food groups by age and sex in all countries, and we applied those model estimates down to individual foods. As an example, the per capita supply of “pumpkins, squash, and gourds” from GENuS (e.g., Cameroon average = ~23.8 g/d) was matched with the relative consumption, by age‐sex group, of “vegetables” from the GDD (25 year old Cameroonian women consume 4.2% less than average) to estimate the relative supply by age‐sex group of individual foods (Cameroonian pumpkin supply for 25 year old women = 22.8 g/d). We assumed that foods in our database that did not belong to a corresponding GDD food group were eaten in proportion to the age‐sex groups' average dietary energy requirements. For the example of the Cameroonian woman, because the GDD has no entry for roots and tubers, her cassava supply was allocated based on her dietary energy needs (national average calorie supply = 2175; estimated calorie needs of a 25 year old Cameroonian woman = 2200, 0.9% greater than national average; cassava supply for 25 year old woman = 218.8 g/d, 0.9% greater than the national average of 216.8 g/d). Each of the 225 foods was then matched with corresponding foods from regional nutrient density tables to estimate dietary iron. Uncertainties from the GDD models of food intakes by age‐sex category were propagated using Monte Carlo sampling of each uncertainty distribution taking 1000 random draws.

We then examined the impact of eCO_2_ on the iron content of diets. To do this, we used the metaanalysis performed by *Myers et al.* [2014], which calculated the percentage change in iron content of edible portions of foods when grown under eCO_2_. In the Myers et al. study, the percentage change in iron content was calculated for several specific crops (e.g., wheat, maize, and field peas), which were also assigned to broader categories based on plant type and photosynthetic pathway. In our study, we calculated percentage change to the specific crops and calculated the change for each broader category (C_3_ grasses and legumes) using a weighted average based on the number of samples collected and analyzed, similar to a subsequent analysis by *Myers et al.* [2015]. A skew‐normal distribution was derived for each food and broader category based on the uncertainty intervals provided by *Myers et al.* [2014], and Monte Carlo simulations (*n* = 1000) were also run on the range of potential eCO_2_‐related iron losses from all foods. Sorting food groups according to this categorization method, as well as their associated average percentage changes in iron, are shown in Tables S1 and S2 in the supporting information. Sorghum and potato samples showed no significant change in iron content when grown under high‐CO_2_ conditions (*P* value: 0.153 [sorghum]; 0.555 [potato]) and the percentage change was assumed to be zero.

To estimate total per capita change in dietary iron under eCO_2_ and subsequent vulnerability, we multiplied the predicted change in iron content by the current dietary iron supply for each food and summed them on each iteration of the Monte Carlo simulation, and then mapped this potential effect on the current landscape of iron deficiency, represented by the WHO global prevalence of anemia for 2011 [*WHO*, 2011]. Anemia may be caused by many factors besides iron deficiency, including parasitic infections, vitamin A deficiency, vitamin B12 deficiency, folate deficiency, and inflammation [*WHO*, 2001; *WHO‐CDC*, 2004; *Nguyen et al*., 2015; *Raiten et al*., 2015]. However, anemia is the most common clinical expression of iron deficiency and provides its best global proxy, despite the common acknowledgement that there are many more people globally suffering from moderate iron deficiency that are not anemic but are still suffering adverse effects. Because of the offsetting effects of underestimation (by not capturing those that are not yet anemic but still iron deficient) and overestimation (by neglecting the multiple etiologies of anemia besides iron deficiency), anemia provides an imperfect stand‐in for iron deficiency. Nevertheless, the prevalence of anemia remains our sole global proxy for iron deficiency, yet we treat it with caution and only use it as a broad relative indicator to estimate countries that may be suffering from iron deficiency and may be most vulnerable to future losses.

Finally, risk categories were established based on two criteria. The first was based on current measured WHO classifications of the prevalence of anemia: greater than 20% anemic (classified as “moderate” or “severe”) or less than 20% (classified “mild”). The second criterion used was the percentage of dietary iron lost under eCO_2_. To establish relative grades of risk based on our results, we separated countries into tertiles, with the cutoffs between categories chosen based on the distribution of estimated iron losses in all countries: >3.8%, 3.1–3.8%, and <3.1%.

**Figure 1:**
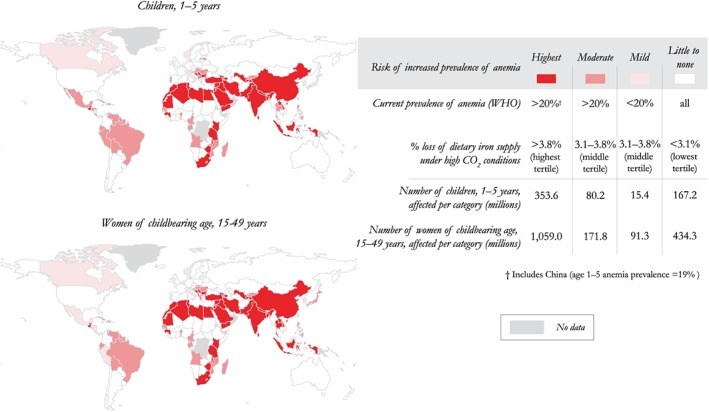
Risk of greater health burden from iron deficiency due to dietary changes for crops grown under high CO_2_ conditions. Highest risk categories (dark red) included populations currently suffering from >20% prevalence of anemia and who also saw a greater than 3.8% loss of dietary iron supply under eCO_2_ conditions. Moderate risk (salmon red) populations currently have a prevalence of anemia greater than 20%, while also potentially losing between 3.1 and 3.8% under eCO_2_. Mild risk countries (light pink) also have a projected loss of dietary iron supply in the middle tertile (3.1–3.8%) and experience a current prevalence of anemia below 20%. Countries with little to no risk of increased vulnerability (white) are projected to have a loss of dietary iron supply in the lowest tertile (<3.1%), regardless of current prevalence of anemia.

Countries with a prevalence of anemia over 20% and iron losses in the highest tertile (>3.8%) were classified “high” risk. Countries with iron losses in the middle tertile were classified “moderate” if they had a prevalence of anemia over 20% and “mild” if they were under 20%. Countries whose iron losses were in the lowest tertile (<3.1%) had a risk classification of “little to none,” regardless of current anemia prevalence (Figure 1). To assess the specific effect on the poorest consumers who are also least likely among the populations to consume animal source foods, we also analyzed the case of vegetarian diets. To do this, we removed all animal source foods from each country's nationally averaged diet (including meat, fish, dairy, and eggs) and replaced those calories with a proportional increase in the remaining vegetal foods, similar to *Myers et al*. [2015].

## 3 Results

We found that the populations in the category at high risk for increased burden of iron deficiency associated with anthropogenic CO_2_ emissions constitute 1.4 billion people (Figure 1) (59% of all children under 5 and women of childbearing age worldwide). That total includes 354 million children age 1–5 (57% of all 1–5 year old children) and 1.06 billion women of childbearing age (60% of all women of childbearing age). Regions with the highest risk were located in South and East Asia, as well as North and East Africa. Populations in the moderate risk category are primarily located in South America and West Africa. Vulnerable groups in these countries total 80 million children (13% of world total) and 172 million women of childbearing age (10% of world total).

Across all countries, the estimated percentages of lost dietary iron under eCO_2_ ranged from modest to more severe: 1.5–5.5%. A full list of predicted iron losses for each country, as well as their current prevalence of anemia and our assigned risk category may be found in Tables S3 (children) and S4 (women). These losses were heterogeneously distributed globally, with the largest losses located in India, the Middle East, and North Africa.

**Figure 2:**
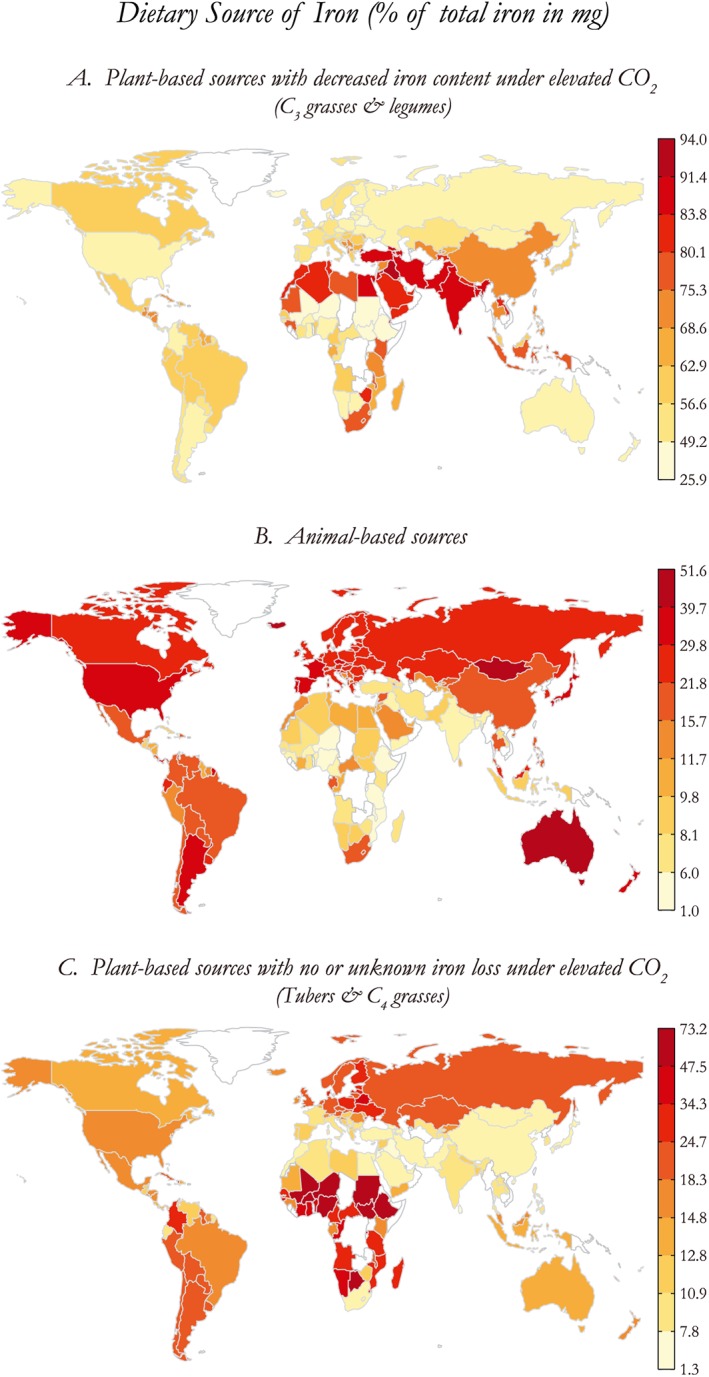
Percentage of total dietary iron sourced from different foods.

The reasons for this heterogeneity are explored in two ways by examining which foods and diets were preferentially leading to iron vulnerability under eCO_2_. The first, shown in Figure 2, shows the percentage of total dietary iron derived from different food sources by country for both children and women of childbearing age. As expected, countries that gain most of their iron from the foods that are influenced by the eCO_2_ effect (Figure 2a) are also those most vulnerable to future iron declines shown in Figure 1. The second, listed in Table 1, identifies the top sources of lost iron from eCO_2_, summed across all populations in the highest‐risk countries defined in Figure 1. Here we find that over 50% of the lost iron among high‐risk groups is derived from several cereal grains and flours (wheat, wheat flour, rice, maize, and corn flour) while another 18% is sourced from “vegetables, nes,” driven predominantly by very high consumption in China, despite a low‐to‐moderate iron content. The remaining 30% is derived from a wider assortment of legumes, vegetables, nuts, and fruits.

**Table 1:** Top Dietary Sources of Lost Iron Under eCO_2_ a

Food	Aggregate Daily Iron Lost Under eCO_2_ Among High‐Risk Populations (kg/d)
Wheat	290.8
Vegetables, fresh, nes	235.4
Rice	172.7
Wheat flour	157.7
Maize	46.8
Cabbages and other brassicas	31.4
Soyabeans	24.3
Beans	20.2
Tomatoes	20.0
Corn flour	19.8

a The largest food sources that see the most iron lost under eCO_2_ conditions are grains and cereal flours. The remaining losses are derived from an assortment of smaller contributions from legumes, vegetables, fruits, and nuts.

In contrast to the high‐risk countries, the countries that derive most of their iron from animal‐based sources (Figure 2b) are generally among the least vulnerable, tending to be wealthier and less anemic: North America, Europe, Australia, and New Zealand. There are also many countries in sub‐Saharan Africa and South America that derive a majority of their iron from plant‐based sources that receive no or an unknown effect on their iron content when grown under high‐CO_2_ conditions (tubers, C_4_ plants excluding maize and aquatic plants) and therefore see a smaller increase in vulnerability (Figure 2c).

**Figure 3:**
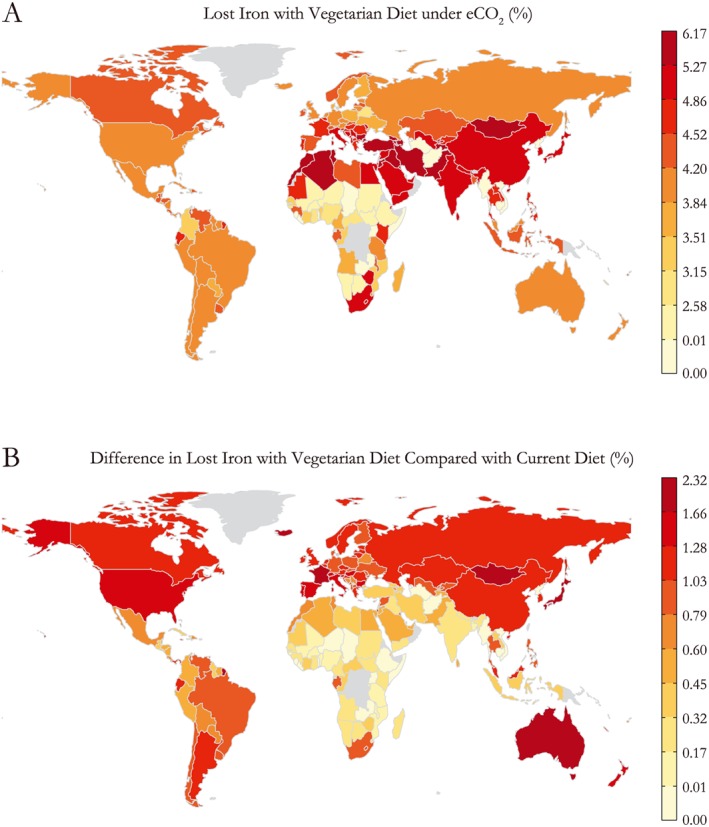
Lost dietary iron under eCO_2_ for vegetarian diets in each country. Vegetarian diets were estimated by removing animal source foods from the diet and replacing them with a proportional increase in the remaining vegetal portion of the diet to maintain caloric supply.

**Figure 4:**
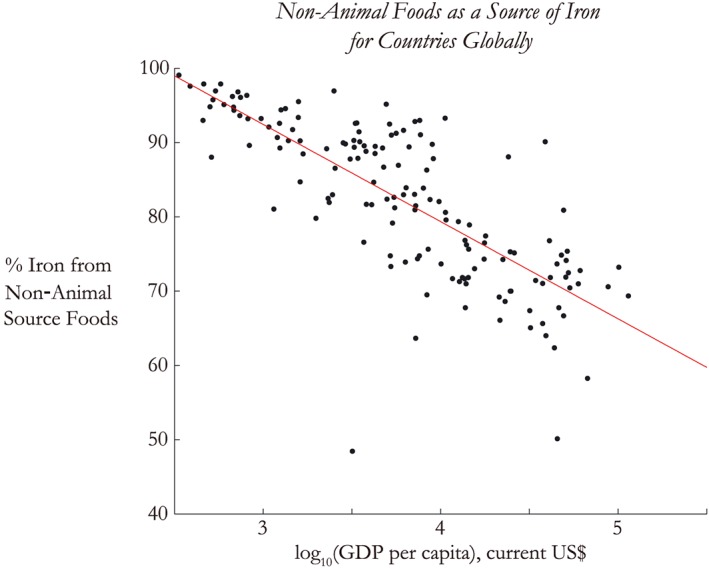
Percentage of iron derived from nonanimal source foods is correlated with income, shown here as the logarithm of GDP per capita [*FAO*, 2016]. Poorer countries generally derive less iron from animal source foods than wealthier ones, putting them at higher risk of iron deficiency due to the lower bioavailability and absorption of vegetal nonheme‐type iron.

We also analyzed vegetarian diets by country and their resulting dietary iron supply (described further section 2 above), we find that the incremental decrease (in excess of the national‐average diet) in dietary iron may be as large as an additional 2.3% in regions that typically consume a large amount of meat: Central Asia, Europe, and the U.S. (Figure 3). Furthermore, a wide range of vegetarian diets within larger middle‐income countries could see a considerable decrease in dietary iron as a result of eCO_2_: Argentina, Brazil, South Africa, Malaysia, Mongolia, and the Philippines. In total, those people consuming vegetarian diets in China, India, and much of the Middle East and South Asia could experience greater than 5% losses of dietary iron due to the eCO_2_ effect. Given the strong correlation between income and animal source food (ASF) consumption (Figure 4), we suspect that poorer populations within countries may be more likely to consume a vegetarian diet. We believe this trend would hold for rural communities in low‐income countries as well, where livestock ownership is consistent regardless of income, yet the poorest smallholders sell a greater proportion of their animal products rather than consume them [*FAO*, 2012]. Consequentially, those populations could potentially be exposed to these higher rates of dietary iron loss.

## 4 Discussion

The geographic and demographic distribution of those most vulnerable to the impact of eCO_2_ on dietary iron is illuminating. First, vulnerable countries are quite often among the poorest globally and therefore least likely to have the financial means to “buy” their way out of this problem via relatively expensive animal‐sourced foods. Furthermore, the poorest populations in already low‐income countries that consume vegetarian diets may also see appreciable declines beyond those seen on average nationally, up to an extra 2% in lost dietary iron. In fact, the lack of animal sources for dietary iron also creates a greater imbalance in iron status due to the type of iron consumed. Animal source foods contain heme iron, which is more easily absorbed by the body compared to nonheme iron, which constitutes the entirety of iron coming from vegetal sources. Generally, ~15–40% of heme iron will be absorbed by the body, while absorption of nonheme iron can range from 1 to 15% [*Hunt*, 2002], depending on an individual's iron stores, health status, and nutrients eaten alongside the iron [*Hallberg and Hulthen*, 2000; *Armah et al*., 2012]. Nonheme iron absorption may also be lower in some low‐income countries where anemia is prevalent due to concurrent parasitic disease, inflammation, malnutrition, and phytate intake, all of which can inhibit absorption [*Zimmermann and Hurrell*, 2007]. Because eCO_2_ affects only nonheme iron supply, it disproportionately impacts populations who rely most heavily on nonheme sources for their dietary iron. These populations are those who also tend to have the fewest resources to overcome this vulnerability and are most afflicted with concurrent exacerbating parasitic, inflammatory, and malnutrition‐related disease. This relationship is shown in Figure 4, which demonstrates the greater reliance of poorer countries (as measured by GDP per capita) on nonanimal sources for iron, which supply only nonheme iron.

**Figure 5:**
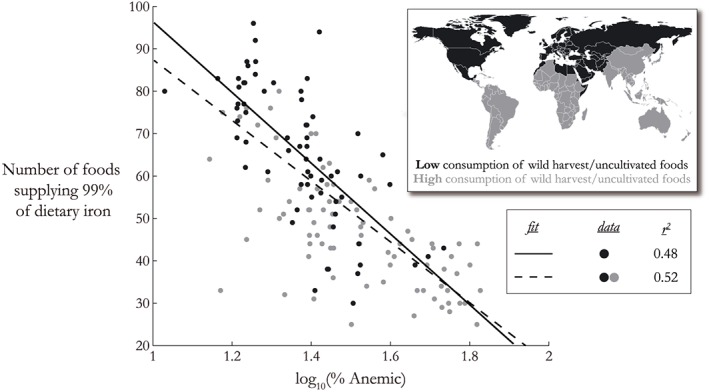
Number of iron sources that constitute 99% of the each country's dietary iron, compared with the current prevalence of anemia for vulnerable groups (children aged 1–5 and women of childbearing age). Black dots indicate countries that consume a low amount of wild harvest/uncultivated foods, grey dots represent countries with high consumption (identified by *Bharucha and Pretty* [2010]); country categorization is shown in the inset map. Countries suffering with the highest prevalence of anemia also have the fewest iron sources among cultivated foods to compensate for any losses. Even when restricting the regression to countries with low consumption of wild harvest/uncultivated foods, this trend is still observed.

Another implication of the relationship between wealth, anemia, and the predicted loss of iron in eCO_2_ conditions is that many of the most affected countries may also have a lower diversity of dietary iron sources from cultivated foods, obstructing a transition to other iron‐rich sources to compensate for any potential losses. This is shown more explicitly in Figure 5, where we have examined the number of foods that provide 99% of the iron in the diet. We calculated it in this way to exclude foods consumed in very small (≪1%) proportions that do not reflect usual diets. At a national level, diversity of dietary iron sources and current prevalence of anemia are negatively correlated, where countries with the highest prevalence of anemia are receiving their dietary iron from the fewest cultivated sources, and vice versa. Similarly, there is a logarithmic relationship between them, indicating that dietary diversity falls off sharply among countries with higher prevalence of anemia, and moderately and severely afflicted countries share a similar number of dietary iron sources.

However, many low‐income countries are more reliant on a variety of wild‐harvest plants and animals to complement cultivated foods in the diet and supply necessary micronutrients. These foods are commonly not captured in statistics provided to the FAO. To examine whether the omission of these types of unreported foods impacts our results, we reexamined the diversity of iron sources after removing the regions of the world that consume high amounts of unaccounted wild or locally grown foods, as identified by *Bharucha and Pretty* [2010]. We find that the trend persists even among those middle‐ and higher‐income countries that are less reliant on wild foods, which suggests that this is a durable result even after excluding locations with incomplete accounting of the full diet.

One limitation of our study was that our analysis was limited only to dietary iron supplies rather than bioavailable iron or iron status (e.g., serum ferritin levels) due to our unconstrained biophysical controls on iron absorption. The quantity of iron absorbed from foods strongly depends on an individual's iron stores (typically measured by serum ferritin) and, to a lesser extent, other foods and nutrients in the diet, which may contain substances that enhance absorption, such as ascorbic acid, or inhibit absorption, such as phytate or calcium [*Hallberg and Hulthen*, 2000; *Zimmermann and Hurrell*, 2007; *Armah et al*., 2012; *Collings et al*., 2013; *Dainty et al*., 2014]. Currently, no global data sets on serum ferritin or other direct iron status measurements exist. Likewise, several substances that affect iron absorption (e.g., tannins, casein, and polyphenols) are not commonly measured for most foods, thereby limiting our ability to predict dietary controls on absorption. Therefore, we relied upon the prevalence of anemia and total annually averaged iron supplies to estimate the potential burden of lowered dietary iron. However, more study is warranted on the accompanying changes in bioavailable iron and iron status to gain a fuller understanding of the projected health implications.

Furthermore, because of the uncertainty in the iron bioavailability of national diets, we are unable to confidently calculate the rise in iron deficiency that would result from our estimated dietary iron losses of 1.5 to 5.5%. However, a companion study that investigated the impact of eCO_2_ on dietary zinc supply and zinc deficiency showed that similarly modest declines may result in 138 million additional people becoming zinc deficient [*Myers et al*., 2015]. Another study analyzing the effect of eCO_2_ on protein estimated a 4.7% global decrease in dietary protein supplies, resulting in an additional 247 million people becoming protein deficient [*Medek et al*., 2017]. From these studies it is plausible that, at minimum, tens of millions of people could become iron deficient as a result of similar losses in dietary iron under eCO_2_.

Another assumption of our model that is likely to impact our results is the presumption of constant diets under a changing atmosphere. Traditional economic models predict that income levels will rise globally through 2050, especially in the developing world. These changes are assumed to drive increases in overall food consumption per capita, particularly of expensive foods that may improve dietary iron such as livestock products [e.g., *Alexendratos and Bruinsma*, 2012]. These models have generally treated climate change as a small‐to‐moderate headwind on overall economic and nutritional improvement [e.g., *Springmann et al*., 2016; *Roson and van der Mensbrugghe*, 2010]. However, recent modeling intercomparisons have examined a suite of potential scenarios for the combined effects of economic growth and climate change on food production and have called the traditional view into question. These predict large uncertainties about the composition of future diets that hinge on assumptions about the rate of global economic growth, future climate impacts on food production and access, and increasing resource scarcity [*Valin et al*., 2014]. From this, it is possible to imagine a world where food production will be able to keep pace with demand and economic growth will improve diets, but it is also possible to imagine a world where food production will not be able to keep up, where food prices will rise in response to more expensive inputs of land, water, and production techniques (e.g., precision agriculture), and where economic growth will be depressed by climate change and diets will become impoverished. Without knowing the trajectory of these key variables, it is doubtful that we can meaningfully predict future diets. Though it is clear that dietary patterns will change over the coming decades, until there is greater certainty in the directionality and magnitude of the dietary impacts from economic and climate shifts, we chose to make the simplest and most transparent assumption of maintaining constant diets.

There are several solutions for countries that disproportionally suffer from reduced dietary iron. Most strategies include a more systematic and dedicated expansion of technology‐driven solutions, such as governmental or industrial food fortification programs, as well as crop biofortification or targeted supplementation or fortification programs (the extent and impact of current fortification programs is discussed in the supporting information). Countries may also explore nutrition‐based programs to empower farmers and consumers to make informed decisions about food choices to combat their specific health challenges. However, as is clear from recent reviews of the efficacy of treating iron deficiency, what is needed is likely a combination of these pathways to build comprehensive treatments to balance both short‐term and long‐term strategies for fighting iron deficiency [*Pasricha et al*., 2013]. Specifically, recent studies advocate for a multipronged approach to treating iron deficiency in low‐income countries, including both an increase in iron‐rich foods as well as simultaneous treatment of diseases that limit iron absorption (malaria, parasitic infections, and inflammation) [*Raiten et al*., 2016]. Therefore, countries that are likely to see an even greater burden from iron deficiency under eCO_2_ have greater incentive to develop and implement a more comprehensive and targeted strategy to combat iron deficiency so as to not only solve today's health crises but also to protect against future harm.

## 5 Conclusions

In this study we find that, without offsetting effects, there are 1.4 billion children (ages 1–5) and women of childbearing age living in regions that are at the highest risk for increased iron deficiency as a result of the elevated CO_2_ levels that are predicted to occur within the coming decades. Of particular concern are poorer subpopulations consuming vegetarian diets in Asia and the Middle East who may rely more heavily on eCO_2_‐affected vegetal sources for their iron. Because these changes will be gradual and largely imperceptible, it will require ongoing monitoring of the nutrient content of their crops, as well as their national nutritional adequacy, to evaluate when and how to most effectively intervene if necessary. Ultimately, global increases in CO_2_ will exert detrimental outcomes for many countries, including many of the poorest, and controlling future CO_2_ emissions will not only help alleviate climatic and biological consequences but avert future health and nutrition impacts as well.

## Conflict of Interest

The authors declare no conflicts of interest relevant to this study.

## Supporting information



Supporting Information S1
