# Supplementary material for: Potential rise in iron deficiency due to future anthropogenic carbon dioxide emissions
Source: Geohealth. 2017 Aug 2;1(6):248–57. doi: 10.1002/2016GH000018 (PMC7007116; doi:10.1002/2016GH000018)
Supplement: Supplementary file 1 — Supporting Information S1 [file GH2-1-248-s001.pdf]

Supporting Information

Supplementary Results

A common strategy to address iron deficiency is through fortification of cereal flours, mainly wheat, with added iron compounds or electrolytic iron. Within the GENuS model, we also attempted to estimate the amount of iron being added through fortification by assembling a database of mandatory and voluntary guidelines of flour fortification, based primarily on the Food Fortification Initiative country surveys [Food Fortification Initiative, 2016a]. Of the 1.4B people living in countries listed as "highest" risk in this study, roughly 21% (293M) live in a country that has a national guideline for fortifying flour, of which 30% reside in Indonesia. Other regions with existing fortification programs, which may be intensified to help meet projected iron shortfalls, are mainly in the Middle East (Egypt, Iran, Saudi Arabia, Jordan, UAE, Kuwait, Morocco), East Africa (Kenya, Tanzania, Djibouti), Central Europe (Albania, Moldova), and Latin America & the Caribbean (Belize, Cuba, Guatemala). For India, the second largest high-risk country in our analysis (below China), though it does not yet have a national or state-level policy on fortification, an estimated 12% of wheat flour is currently being fortified through private partnerships [Food Fortification Initiative, 2016b]. Strengthening the existing fortification programs of at-risk countries, as well as building new programs in countries without them and improving targeting to needy groups, may be useful parts of a broader strategy to address projected losses to iron as a result of the eCO_2 effect. However, clearly additional steps must be taken to address the other determining factors of anemia: infectious diseases, inflammatory conditions, and malnutrition.

Food Fortification Initiative, Country fortification profiles (2016a), Available: http://www.ffinetwork.org/country_profiles/
Food Fortification Initiative, Country profile — India (2016b), Available: http://www.ffinetwork.org/country_profiles/country.php?record=100

Supplementary Table Captions

Table S1. Crops categorization by photosynthetic type for modeling iron decreases

Table S2. Average, 95% CI, and number of samples measured (N) for decrease in iron content for individual crops (taken from *Myers et al.* [2014]) and within photosynthetic groups.

Table S3. Median and 95% UI modeled loss in dietary iron under eCO_2 for children ages 0-5, as well as their current rate of anemia [*WHO*, 2011] and assigned risk category (between 0 and 3) for all countries.

Table S4. Median and 95% UI modeled loss in dietary iron under eCO_2 for women of childbearing age (15-49), as well as their current rate of anemia (*WHO,* 2011) and assigned risk category (between 0 and 3) for all countries.

TABLE S1

Crop Type:	*C3 Grasses*	*C3 Legumes*
Crops:	Wheat	Field Peas
	Barley	Soybeans
	Wheat flour	Beans
	Buckwheat	Broad beans; horse beans; dry
	Triticale	Chick peas
	Mixed grain	Cow peas; dry
	Popcorn	Pigeon peas
	Quinoa	Lentils
	Sunflowerseed	Bambara beans
	Rape and Mustardseed	Vetches
	Cottonseed	Lupins
	Coconuts - Incl Copra	Pulses; nes
	Sesameseed	Flour of pulses
	Palmkernels	Groundnuts (Shelled Eq)
	Olives	Groundnut Oil
	Sunflowerseed Oil	
	Rape and Mustard Oil	
	Cottonseed Oil	
	Palmkernel Oil	
	Palm Oil	
	Coconut Oil	
	Sesameseed Oil	
	Olive Oil	
	Tomatoes	
	Onions	
	Cabbages and other brassicas	
	Artichokes	
	Asparagus	
	Lettuce and chicory	
	Spinach	
	Cassava leaves	
	Cauliflowers and broccoli	
	Pumpkins; squash; and gourds	
	Cucumbers and gherkins	
	Eggplants (aubergines)	
	Chillies and peppers; green	
	Onions (inc. shallots); green	
	Garlic	
	Leeks; other alliaceous veg.	

Beans; green
Peas; green
Leguminous vegetables; nes
String beans
Carrots and turnips
Okra
Maize; green
Mushrooms and truffles
Chicory roots
Vegetables; fresh; nes
Vegetables; dried; nes
Vegetables; dehydrated
Vegetables in vinegar
Vegetables; preserved; nes
Vegetables; frozen
Vegetables in tem. preservatives
Vegetables prepared or preserved; frozen
Homogenous vegetables prepared
Watermelons
Other melons (inc. cantaloupes)
Coffee substitutes; cont. coffee
Oranges; Mandarines
Lemons; Limes
Grapefruit
Citrus; Other
Bananas
Plantains
Apples
Dates
Grapes
Pears
Quinces
Apricots
Sour cherries
Cherries
Peaches and nectarines
Plums and sloes
Stone fruit; nes
Pome fruit; nes
Strawberries
Raspberries
Gooseberries

Currants
Blueberries
Cranberries
Berries; nes
Figs
Mangos; mangosteens; guavas
Avocados
Persimmons
Cashewapple
Kiwi fruit
Papayas
Fruit; tropical fresh; nes
Fresh fruit; nes
Fruit dried; nes
Fruit juice; nes
Fruit; prepared; nes
Homogenized; cooked fruit prepared
Coffee
Cocoa Beans
Tea

TABLE S2

Category	*Crop*	*% change in iron at 550 ppm CO_2*	*95% CI*	*N (number of pairs)*
C3 grasses				
	Wheat	-5.9	(-7.8, -4.0)	78
	Rice	-4.9	(-7.3, -2.6)	32
	Barley	-10	(-12.4,-7.4)	6
	**Weighted Average**	**-5.7**	**(-10.3, -3.3)**	
C3 legumes				
	Field peas	-4.1	(-6.7,-1.4)	10
	Soybeans	-5.2	(-7.9,-2.5)	28
	**Weighted Average**	**-4.8**	**(-7.7, -2.1)**	
C4 grasses				
	Maize	-5.8	(-10.9,-0.3)	4

TABLE S3

COUNTRY	ISO3	AGE GROUP<br>MEASURE<br>VALUE	**Children 0-4**<br>Change in Iron with high CO2<br>Median	**Children 0-4**<br>Change in Iron with high CO2<br>Low 95% UI	**Children 0-4**<br>Change in Iron with high CO2<br>High 95% UI	**Children 0-4**<br>Rate of anemia (WHO, 2011)<br>%	**Children 0-4**<br>Risk category<br>Qualitative (0-3)
Afghanistan	AFG		*	*	*	44	*
Albania	ALB		-4.1%	-5.6%	-3.0%	22	3
Algeria	DZA		-5.0%	-6.0%	-4.2%	32	3
Angola	AGO		-3.5%	-4.5%	-2.5%	52	2
Antigua and Barbuda	ATG		-2.8%	-5.1%	-1.2%	38	0
Argentina	ARG		-2.4%	-4.6%	-1.0%	21	0
Armenia	ARM		-4.7%	-6.0%	-3.5%	34	3
Australia	AUS		-2.4%	-3.4%	-1.5%	15	0
Austria	AUT		-3.0%	-4.0%	-2.3%	14	0
Azerbaijan	AZE		-5.0%	-6.5%	-3.8%	35	3
Bahamas	BHS		-3.2%	-4.6%	-2.3%	33	2
Bangladesh	BGD		-4.3%	-5.9%	-3.0%	56	3
Barbados	BRB		-3.2%	-4.1%	-2.4%	37	2
Belarus	BLR		-2.0%	-2.9%	-1.3%	25	0
Belgium	BEL		-3.4%	-4.8%	-2.5%	13	1
Belize	BLZ		-3.8%	-5.7%	-1.8%	35	3
Benin	BEN		-3.2%	-4.5%	-1.9%	65	2
Bermuda	BMU		*	*	*	*	*
Bolivia (Plurinational State of)	BOL		-3.2%	-5.3%	-1.7%	56	2
Bosnia and Herzegovina	BIH		-4.3%	-5.4%	-3.2%	28	3
Botswana	BWA		-2.3%	-3.2%	-1.5%	43	0
Brazil	BRA		-3.1%	-4.9%	-1.6%	24	0
Brunei Darussalam	BRN		-3.1%	-4.5%	-2.3%	18	1
Bulgaria	BGR		-4.3%	-5.2%	-3.4%	27	3
Burkina Faso	BFA		-2.3%	-3.4%	-1.1%	86	0
Cabo Verde	CPV		-4.0%	-5.2%	-2.7%	60	3
Cambodia	KHM		*	*	*	55	*
Cameroon	CMR		-3.4%	-4.4%	-2.4%	63	2
Canada	CAN		-3.4%	-4.4%	-2.6%	14	1
Central African Republic	CAF		-2.7%	-4.0%	-1.6%	72	0
Chad	TCD		*	*	*	74	*
Chile	CHL		-3.0%	-5.1%	-1.3%	21	0
China	CHN		-4.0%	-6.0%	-2.9%	19	3
Colombia	COL		-2.7%	-4.5%	-1.4%	28	0
Congo	COG		-3.0%	-3.6%	-2.6%	65	0
Costa Rica	CRI		-3.3%	-5.5%	-1.4%	33	2
Croatia	HRV		-3.5%	-4.7%	-2.7%	27	2
Cote d'Ivoire	CIV		-2.9%	-4.0%	-2.0%	75	0
Cuba	CUB		-3.7%	-5.7%	-1.8%	34	2
Cyprus	CYP		-3.2%	-4.3%	-2.4%	19	1

Czech Republic	CZE	-2.8%	-3.5%	-2.1%	27	0
Democratic People's Republic of Korea	PRK	*	*	*	34	*
Denmark	DNK	-2.6%	-3.7%	-1.9%	14	0
Djibouti	DJI	-4.6%	-5.5%	-3.6%	43	3
Dominica	DMA	*	*	*	37	*
Dominican Republic	DOM	-3.4%	-5.2%	-1.7%	33	2
Ecuador	ECU	-3.2%	-5.2%	-1.8%	40	2
Egypt	EGY	-4.6%	-5.7%	-3.7%	45	3
El Salvador	SLV	-3.3%	-5.8%	-1.3%	30	2
Estonia	EST	-3.1%	-3.8%	-2.3%	26	0
Ethiopia	ETH	-1.9%	-3.4%	-1.3%	50	0
Fiji	FJI	-3.7%	-4.9%	-2.8%	32	3
Finland	FIN	-2.6%	-3.3%	-2.0%	14	0
France	FRA	-2.9%	-4.0%	-2.0%	14	0
French Polynesia	PYF	-3.1%	-4.2%	-2.3%	*	0
Gabon	GAB	-3.6%	-4.6%	-2.9%	60	2
Gambia	GMB	-2.3%	-3.1%	-1.7%	65	0
Georgia	GEO	-5.0%	-6.4%	-3.8%	26	3
Germany	DEU	-2.9%	-3.9%	-2.2%	14	0
Ghana	GHA	-2.6%	-3.6%	-1.8%	76	0
Greece	GRC	-3.6%	-5.0%	-2.7%	15	1
Grenada	GRD	-3.6%	-4.7%	-2.9%	40	2
Guatemala	GTM	-3.8%	-6.6%	-1.5%	47	3
Guinea-Bissau	GNB	-2.8%	-3.9%	-1.9%	71	0
Guinea	GIN	-4.1%	-5.3%	-3.0%	76	3
Guyana	GUY	-3.5%	-5.3%	-1.7%	41	2
Haiti	HTI	-3.4%	-5.2%	-2.1%	62	2
Honduras	HND	-3.7%	-6.5%	-1.4%	39	2
Hungary	HUN	-3.4%	-4.4%	-2.5%	27	2
Iceland	ISL	-1.9%	-2.9%	-1.1%	14	0
India	IND	-4.8%	-6.3%	-3.7%	59	3
Indonesia	IDN	-4.0%	-5.5%	-3.0%	32	3
Iran (Islamic Republic of)	IRN	-5.0%	-6.2%	-3.9%	32	3
Iraq	IRQ	-5.4%	-6.8%	-4.2%	36	3
Ireland	IRL	-3.1%	-5.0%	-2.3%	14	0
Israel	ISR	-3.6%	-4.7%	-2.7%	15	1
Italy	ITA	-3.6%	-5.0%	-2.7%	14	1
Jamaica	JAM	-3.2%	-4.3%	-2.3%	32	2
Japan	JPN	-3.5%	-4.5%	-2.7%	15	1
Jordan	JOR	-4.9%	-6.1%	-3.8%	31	3
Kazakhstan	KAZ	-3.1%	-3.9%	-2.3%	30	0
Kenya	KEN	-4.3%	-5.9%	-2.6%	46	3
Kiribati	KIR	*	*	*	37	*
Kuwait	KWT	-4.2%	-5.3%	-3.3%	26	3
Kyrgyzstan	KGZ	-3.6%	-4.6%	-2.8%	36	2
Lao People's Democratic Republic	LAO	-4.3%	-6.0%	-3.0%	42	3

Latvia	LVA	-3.2%	-4.0%	-2.4%	26	2
Lebanon	LBN	-4.6%	-5.7%	-3.5%	24	3
Lesotho	LSO	*	*	*	48	*
Liberia	LBR	*	*	*	72	*
Libya	LBY	-4.0%	-5.5%	-2.8%	30	3
Lithuania	LTU	-3.0%	-3.7%	-2.3%	26	0
Luxembourg	LUX	-3.0%	-4.1%	-2.2%	13	0
Madagascar	MDG	-3.5%	-4.8%	-2.4%	50	2
Malawi	MWI	-4.3%	-6.5%	-2.0%	66	3
Malaysia	MYS	-3.0%	-4.3%	-2.3%	32	0
Maldives	MDV	-4.0%	-5.2%	-3.0%	30	3
Mali	MLI	-1.9%	-2.7%	-1.1%	80	0
Malta	MLT	-3.5%	-4.8%	-2.6%	14	1
Mauritania	MRT	-4.1%	-5.0%	-3.3%	71	3
Mauritius	MUS	-4.7%	-5.6%	-4.0%	44	3
Mexico	MEX	-3.3%	-6.3%	-1.2%	26	2
Mongolia	MNG	-3.0%	-4.7%	-1.8%	26	0
Montenegro	MNE	-3.9%	-5.3%	-3.0%	27	3
Morocco	MAR	-5.4%	-6.4%	-4.5%	35	3
Mozambique	MOZ	-3.4%	-4.7%	-2.0%	66	2
Myanmar	MMR	*	*	*	40	*
Namibia	NAM	-2.2%	-3.3%	-1.3%	49	0
Nepal	NPL	-4.6%	-5.7%	-3.6%	51	3
Netherlands Antilles	NDA	*	*	*	*	*
Netherlands	NLD	-2.8%	-4.8%	-2.1%	14	0
New Caledonia	NCL	-3.1%	-4.2%	-2.3%	*	1
New Zealand	NZL	-2.6%	-3.6%	-1.9%	15	0
Nicaragua	NIC	-3.6%	-6.1%	-1.5%	19	1
Niger	NER	-1.5%	-2.1%	-0.9%	76	0
Nigeria	NGA	-2.6%	-3.5%	-1.8%	71	0
Norway	NOR	-3.2%	-4.0%	-2.5%	14	1
Occupied Palestinian Territory	PSE	-4.0%	-5.4%	-3.1%	*	0
Pakistan	PAK	-4.9%	-6.2%	-3.8%	61	3
Panama	PAN	-2.7%	-4.5%	-1.1%	32	0
Paraguay	PRY	-3.0%	-5.5%	-1.4%	44	0
Peru	PER	-3.4%	-5.0%	-2.1%	27	2
Philippines	PHL	-3.7%	-5.3%	-2.5%	33	2
Poland	POL	-2.8%	-3.7%	-1.5%	35	0
Portugal	PRT	-3.2%	-4.2%	-2.4%	26	2
Republic of Korea	KOR	-3.7%	-5.2%	-2.7%	15	1
Republic of Moldova	MDA	-4.0%	-5.2%	-2.7%	29	3
Romania	ROU	-3.5%	-4.5%	-2.7%	27	2
Russian Federation	RUS	-2.8%	-3.7%	-2.0%	26	0
Rwanda	RWA	-3.3%	-4.3%	-2.4%	38	2
Saint Kitts and Nevis	KNA	*	*	*	*	*
Saint Lucia	LCA	-2.9%	-3.8%	-2.2%	41	0

Saint Vincent and the Grenadines	VCT	-4.0%	-5.9%	-2.8%	38	3
Samoa	WSM	*	*	*	32	*
Sao Tome and Principe	STP	*	*	*	59	*
Saudi Arabia	SAU	-4.6%	-5.7%	-3.6%	39	3
Senegal	SEN	-3.2%	-4.1%	-2.2%	79	2
Serbia	SRB	-4.2%	-5.2%	-3.4%	26	3
Sierra Leone	SLE	*	*	*	74	*
Slovakia	SVK	-3.4%	-4.5%	-2.6%	27	2
Slovenia	SVN	-3.3%	-4.2%	-2.4%	27	2
Solomon Islands	SLB	*	*	*	40	*
Somalia	SOM	*	*	*	57	*
South Africa	ZAF	-4.4%	-6.3%	-2.4%	41	3
Spain	ESP	-2.9%	-4.0%	-2.2%	14	0
Sri Lanka	LKA	-4.6%	-6.4%	-3.5%	36	3
Sudan (former)	SDN	-1.9%	-2.5%	-1.4%	59	0
Suriname	SUR	-3.4%	-5.4%	-1.3%	39	2
Swaziland	SWZ	-3.7%	-5.2%	-2.2%	44	2
Sweden	SWE	-2.9%	-3.9%	-2.2%	13	0
Switzerland	CHE	-3.1%	-4.2%	-2.2%	14	0
Syrian Arab Republic	SYR	-4.0%	-5.1%	-3.0%	37	3
Tajikistan	TJK	-4.8%	-6.0%	-3.8%	27	3
Thailand	THA	-3.7%	-5.3%	-2.7%	29	2
The former Yugoslav Republic of Macedonia	MKD	-4.0%	-5.1%	-3.0%	22	3
Timor-Leste	TLS	*	*	*	45	*
Togo	TGO	*	*	*	71	*
Trinidad and Tobago	TTO	-3.6%	-4.7%	-2.8%	39	2
Tunisia	TUN	-4.8%	-5.9%	-4.0%	29	3
Turkey	TUR	-5.0%	-6.2%	-3.9%	30	3
Turkmenistan	TKM	*	*	*	32	*
Uganda	UGA	*	*	*	56	*
Ukraine	UKR	-2.8%	-3.5%	-2.1%	27	0
United Arab Emirates	ARE	-4.5%	-5.5%	-3.6%	29	3
United Kingdom	GBR	-2.9%	-3.9%	-2.2%	13	0
United Republic of Tanzania	TZA	-3.8%	-5.1%	-2.5%	61	3
United States of America	USA	-2.7%	-3.7%	-2.1%	6	0
Uruguay	URY	-3.1%	-5.0%	-1.6%	24	0
Uzbekistan	UZB	-4.3%	-5.6%	-3.1%	43	3
Vanuatu	VUT	*	*	*	38	*
Venezuela (Bolivarian Republic of)	VEN	-3.2%	-5.7%	-1.5%	32	2
Viet Nam	VNM	*	*	*	31	*
Yemen	YEM	-4.6%	-5.8%	-3.5%	59	3
Zambia	ZMB	*	*	*	58	*
Zimbabwe	ZWE	-4.6%	-6.8%	-2.1%	59	3

TABLE S4

COUNTRY	ISO3	AGE GROUP<br>MEASURE<br>VALUE	**Women (15-49)**<br>Change in Iron with high CO2<br>Median	**Women (15-49)**<br>Change in Iron with high CO2<br>Low 95% UI	**Women (15-49)**<br>Change in Iron with high CO2<br>High 95% UI	**Women (15-49)**<br>Rate of anemia (WHO, 2011)<br>%	**Women (15-49)**<br>Risk category<br>Qualitative (0-3)
Afghanistan	AFG		*	*	*	33	*
Albania	ALB		-4.1%	-5.7%	-3.0%	22	3
Algeria	DZA		-5.0%	-5.9%	-4.2%	33	3
Angola	AGO		-3.5%	-4.6%	-2.5%	45	2
Antigua and Barbuda	ATG		-2.8%	-5.1%	-1.3%	25	0
Argentina	ARG		-2.4%	-4.6%	-1.0%	16	0
Armenia	ARM		-4.7%	-6.0%	-3.5%	26	3
Australia	AUS		-2.4%	-3.4%	-1.5%	17	0
Austria	AUT		-3.0%	-4.1%	-2.3%	19	0
Azerbaijan	AZE		-5.0%	-6.4%	-3.7%	33	3
Bahamas	BHS		-3.2%	-4.7%	-2.3%	23	2
Bangladesh	BGD		-4.3%	-5.9%	-3.0%	43	3
Barbados	BRB		-3.2%	-4.1%	-2.4%	23	2
Belarus	BLR		-2.0%	-2.9%	-1.3%	22	0
Belgium	BEL		-3.4%	-4.9%	-2.5%	18	1
Belize	BLZ		-3.8%	-5.8%	-1.9%	22	3
Benin	BEN		-3.2%	-4.5%	-1.9%	50	2
Bermuda	BMU		*	*	*	*	*
Bolivia (Plurinational State of)	BOL		-3.2%	-5.2%	-1.7%	32	2
Bosnia and Herzegovina	BIH		-4.2%	-5.4%	-3.1%	24	3
Botswana	BWA		-2.3%	-3.2%	-1.5%	29	0
Brazil	BRA		-3.1%	-4.9%	-1.6%	20	2
Brunei Darussalam	BRN		-3.2%	-4.6%	-2.4%	20	2
Bulgaria	BGR		-4.2%	-5.1%	-3.3%	24	3
Burkina Faso	BFA		-2.3%	-3.4%	-1.2%	50	0
Cabo Verde	CPV		-4.0%	-5.2%	-2.7%	38	3
Cambodia	KHM		*	*	*	44	*
Cameroon	CMR		-3.4%	-4.5%	-2.4%	42	2
Canada	CAN		-3.4%	-4.4%	-2.6%	17	1
Central African Republic	CAF		-2.7%	-4.0%	-1.6%	46	0
Chad	TCD		*	*	*	47	*
Chile	CHL		-3.0%	-5.1%	-1.3%	12	0
China	CHN		-4.1%	-6.1%	-3.0%	20	3
Colombia	COL		-2.6%	-4.5%	-1.4%	19	0
Congo	COG		-3.0%	-3.6%	-2.6%	51	0
Costa Rica	CRI		-3.3%	-5.6%	-1.4%	19	1
Croatia	HRV		-3.5%	-4.8%	-2.6%	25	2
Cote d'Ivoire	CIV		-2.9%	-4.0%	-2.0%	49	0
Cuba	CUB		-3.7%	-5.7%	-1.8%	23	2
Cyprus	CYP		-3.2%	-4.4%	-2.4%	28	2

Czech Republic	CZE	-2.7%	-3.6%	-2.1%	23	0
Democratic People's Republic of Korea	PRK	*	*	*	25	*
Denmark	DNK	-2.6%	-3.8%	-1.9%	18	0
Djibouti	DJI	-4.6%	-5.5%	-3.7%	27	3
Dominica	DMA	*	*	*	25	*
Dominican Republic	DOM	-3.4%	-5.2%	-1.7%	26	2
Ecuador	ECU	-3.3%	-5.3%	-1.8%	24	2
Egypt	EGY	-4.6%	-5.7%	-3.6%	35	3
El Salvador	SLV	-3.3%	-5.8%	-1.4%	23	2
Estonia	EST	-3.0%	-3.8%	-2.3%	24	0
Ethiopia	ETH	-1.9%	-3.4%	-1.3%	19	0
Fiji	FJI	-3.7%	-4.9%	-2.8%	27	2
Finland	FIN	-2.6%	-3.3%	-2.0%	18	0
France	FRA	-2.9%	-4.0%	-2.0%	19	0
French Polynesia	PYF	-1.9%	-2.8%	-1.4%	*	0
Gabon	GAB	-3.6%	-4.7%	-2.9%	51	2
Gambia	GMB	-2.4%	-3.1%	-1.7%	45	0
Georgia	GEO	-5.0%	-6.4%	-3.8%	28	3
Germany	DEU	-2.8%	-3.9%	-2.2%	18	0
Ghana	GHA	-2.6%	-3.6%	-1.8%	56	0
Greece	GRC	-3.6%	-5.1%	-2.7%	20	2
Grenada	GRD	-3.6%	-4.7%	-2.8%	26	2
Guatemala	GTM	-3.8%	-6.6%	-1.5%	26	3
Guinea-Bissau	GNB	-2.8%	-3.8%	-1.9%	45	0
Guinea	GIN	-4.1%	-5.4%	-3.0%	48	3
Guyana	GUY	-3.5%	-5.3%	-1.6%	34	2
Haiti	HTI	-3.4%	-5.2%	-2.1%	37	2
Honduras	HND	-3.7%	-6.4%	-1.4%	18	1
Hungary	HUN	-3.3%	-4.3%	-2.5%	24	2
Iceland	ISL	-1.9%	-3.0%	-1.1%	17	0
India	IND	-4.8%	-6.3%	-3.7%	48	3
Indonesia	IDN	-4.0%	-5.5%	-3.0%	23	3
Iran (Islamic Republic of)	IRN	-5.0%	-6.2%	-3.9%	28	3
Iraq	IRQ	-5.4%	-6.8%	-4.2%	31	3
Ireland	IRL	-3.1%	-5.0%	-2.3%	17	0
Israel	ISR	-3.6%	-4.8%	-2.7%	17	1
Italy	ITA	-3.5%	-5.1%	-2.6%	19	1
Jamaica	JAM	-3.2%	-4.3%	-2.3%	24	2
Japan	JPN	-3.5%	-4.5%	-2.7%	22	2
Jordan	JOR	-4.9%	-6.1%	-3.8%	28	3
Kazakhstan	KAZ	-3.1%	-4.0%	-2.3%	30	0
Kenya	KEN	-4.3%	-5.9%	-2.6%	25	3
Kiribati	KIR	*	*	*	21	*
Kuwait	KWT	-4.2%	-5.4%	-3.3%	22	3
Kyrgyzstan	KGZ	-3.7%	-4.7%	-2.8%	32	2
Lao People's Democratic Republic	LAO	-4.2%	-6.0%	-3.0%	31	3

Latvia	LVA	-3.1%	-3.9%	-2.3%	24	0
Lebanon	LBN	-4.5%	-5.7%	-3.5%	27	3
Lesotho	LSO	*	*	*	27	*
Liberia	LBR	*	*	*	49	*
Libya	LBY	-4.0%	-5.5%	-2.8%	28	3
Lithuania	LTU	-2.9%	-3.7%	-2.3%	23	0
Luxembourg	LUX	-3.0%	-4.2%	-2.2%	18	0
Madagascar	MDG	-3.4%	-4.7%	-2.4%	32	2
Malawi	MWI	-4.3%	-6.4%	-2.0%	29	3
Malaysia	MYS	-3.0%	-4.4%	-2.3%	21	0
Maldives	MDV	-4.0%	-5.2%	-3.0%	37	3
Mali	MLI	-1.9%	-2.7%	-1.1%	56	0
Malta	MLT	-3.5%	-4.9%	-2.6%	17	1
Mauritania	MRT	-4.1%	-5.0%	-3.3%	39	3
Mauritius	MUS	-4.7%	-5.6%	-3.9%	23	3
Mexico	MEX	-3.2%	-6.3%	-1.2%	14	1
Mongolia	MNG	-3.0%	-4.8%	-1.8%	20	0
Montenegro	MNE	-3.9%	-5.3%	-3.0%	24	3
Morocco	MAR	-5.4%	-6.3%	-4.5%	33	3
Mozambique	MOZ	-3.4%	-4.7%	-2.0%	44	2
Myanmar	MMR	*	*	*	30	*
Namibia	NAM	-2.2%	-3.3%	-1.3%	33	0
Nepal	NPL	-4.6%	-5.7%	-3.6%	36	3
Netherlands Antilles	NDA	*	*	*	*	*
Netherlands	NLD	-2.8%	-4.8%	-2.0%	18	0
New Caledonia	NCL	-1.9%	-2.7%	-1.4%	*	0
New Zealand	NZL	-2.6%	-3.6%	-1.9%	15	0
Nicaragua	NIC	-3.6%	-6.1%	-1.5%	13	1
Niger	NER	-1.5%	-2.2%	-0.9%	47	0
Nigeria	NGA	-2.6%	-3.5%	-1.8%	49	0
Norway	NOR	-3.2%	-4.0%	-2.5%	17	1
Occupied Palestinian Territory	PSE	-4.0%	-5.5%	-3.1%	*	0
Pakistan	PAK	-4.9%	-6.2%	-3.8%	51	3
Panama	PAN	-2.7%	-4.5%	-1.0%	25	0
Paraguay	PRY	-2.9%	-5.5%	-1.4%	19	0
Peru	PER	-3.4%	-5.0%	-2.1%	18	1
Philippines	PHL	-3.7%	-5.3%	-2.5%	25	2
Poland	POL	-2.8%	-3.7%	-1.5%	23	0
Portugal	PRT	-3.2%	-4.2%	-2.3%	19	1
Republic of Korea	KOR	-3.7%	-5.3%	-2.7%	19	1
Republic of Moldova	MDA	-3.9%	-5.1%	-2.7%	26	3
Romania	ROU	-3.5%	-4.5%	-2.6%	24	2
Russian Federation	RUS	-2.8%	-3.6%	-2.0%	21	0
Rwanda	RWA	-3.3%	-4.3%	-2.4%	17	1
Saint Kitts and Nevis	KNA	*	*	*	*	*
Saint Lucia	LCA	-2.9%	-3.9%	-2.2%	26	0

Saint Vincent and the Grenadines	VCT	-4.0%	-5.9%	-2.8%	25	3
Samoa	WSM	*	*	*	18	*
Sao Tome and Principe	STP	*	*	*	43	*
Saudi Arabia	SAU	-4.6%	-5.8%	-3.6%	40	3
Senegal	SEN	-3.2%	-4.2%	-2.2%	57	2
Serbia	SRB	-4.2%	-5.2%	-3.3%	25	3
Sierra Leone	SLE	*	*	*	45	*
Slovakia	SVK	-3.4%	-4.6%	-2.6%	23	2
Slovenia	SVN	-3.2%	-4.2%	-2.3%	24	2
Solomon Islands	SLB	*	*	*	25	*
Somalia	SOM	*	*	*	43	*
South Africa	ZAF	-4.4%	-6.3%	-2.4%	28	3
Spain	ESP	-2.9%	-4.0%	-2.1%	18	0
Sri Lanka	LKA	-4.6%	-6.5%	-3.5%	26	3
Sudan (former)	SDN	-1.9%	-2.6%	-1.4%	31	0
Suriname	SUR	-3.4%	-5.5%	-1.4%	25	2
Swaziland	SWZ	-3.7%	-5.1%	-2.2%	28	2
Sweden	SWE	-2.9%	-3.9%	-2.1%	18	0
Switzerland	CHE	-3.1%	-4.2%	-2.1%	19	0
Syrian Arab Republic	SYR	-4.0%	-5.1%	-3.0%	31	3
Tajikistan	TJK	-4.8%	-6.0%	-3.8%	25	3
Thailand	THA	-3.7%	-5.3%	-2.7%	24	2
The former Yugoslav Republic of Macedonia	MKD	-4.0%	-5.2%	-3.0%	19	0
Timor-Leste	TLS	*	*	*	22	*
Togo	TGO	*	*	*	53	*
Trinidad and Tobago	TTO	-3.6%	-4.7%	-2.8%	25	2
Tunisia	TUN	-4.8%	-5.8%	-3.9%	28	3
Turkey	TUR	-5.0%	-6.2%	-4.0%	29	3
Turkmenistan	TKM	*	*	*	32	*
Uganda	UGA	*	*	*	27	*
Ukraine	UKR	-2.7%	-3.5%	-2.1%	23	0
United Arab Emirates	ARE	-4.5%	-5.6%	-3.6%	26	3
United Kingdom	GBR	-2.9%	-4.0%	-2.1%	15	0
United Republic of Tanzania	TZA	-3.8%	-5.1%	-2.5%	40	3
United States of America	USA	-2.7%	-3.7%	-2.1%	12	0
Uruguay	URY	-3.0%	-4.9%	-1.5%	17	0
Uzbekistan	UZB	-4.3%	-5.6%	-3.1%	52	3
Vanuatu	VUT	*	*	*	22	*
Venezuela (Bolivarian Republic of)	VEN	-3.2%	-5.7%	-1.6%	22	2
Viet Nam	VNM	*	*	*	14	*
Yemen	YEM	-4.6%	-5.7%	-3.5%	38	3
Zambia	ZMB	*	*	*	29	*
Zimbabwe	ZWE	-4.5%	-6.8%	-2.1%	28	3
